# Myocardial involvement in the hemodynamic abnormalities associated with acute rheumatic fever

**DOI:** 10.4103/0975-3583.74260

**Published:** 2010

**Authors:** Gamela Nasr, Badr Mesbah, Alaa Saad

**Affiliations:** *Department of Cardiology, Suez Canal University, Ismailia, Egypt*; 1 *Department of Pediatrics, Suez Canal University, Ismailia, Egypt*; 2 *Department of Clinical Pathology, Suez Canal University, Ismailia, Egypt*

**Keywords:** Acute rheumatic fever, cardiac troponin assay, echocardiography, myocardial involvement

## Abstract

**Background and Aim:**

Rheumatic fever is still a common cause of acquired heart disease in children and young adult in many developing countries. The aim was to investigate the role of myocardial involvement in the hemodynamic changes in patients with acute rheumatic fever using cardiac troponin assay and echocardiography. Design: A prospective cohort study was designed.

**Patients and Methods:**

Thirty-four children with acute rheumatic fever, 20 with carditis, and 14 without carditis. Level of cardiac troponin T (cTnT) and echocardiographic measurement of left ventricular function were the main outcome measure.

**Results:**

The level of cardiac troponin in children with carditis was 0.051 ± 0.01 ng/dL, and it was 0.039 ± 0.02 ng/dL in those without carditis. The difference is not significant. In addition, there was no significant difference between the children with carditis and those without carditis regarding left ventricular ejection and shortening fractions.

**Conclusion:**

There are no significant echocardiographic measurements abnormalities or cTnT levels elevation suggesting clinically relevant hemodynamic abnormalities due to myocardial involvement during acute rheumatic fever.

## INTRODUCTION

Rheumatic fever is the most common cause of acquired heart disease in children and young adults all over the world.[1] Despite a documented decrease in the incidence of acute rheumatic fever and a similar documented decrease in the prevalence of rheumatic heart diseases in industrialized countries during the past five decades, these non-suppurative cardiovascular sequelae of group *Streptococcal pharyngitis* remain medical and public health problems in both industrialized and industrializing countries even at the beginning of the 21st century. The most devastating effects are on children and young adults in their most productive years.[2] Rheumatic carditis may be associated with pancarditis involving active inflammation of endocardium, myocardium, and pericardium.[3] Although the pathological specimens show interstitial myocardial infiltrations and Aschoff nodules, myocyte necrosis has not been shown even in the presence of congestive heart failure. Myocyte necrosis is an important finding in the diagnosis of myocarditis.[4] Cardiac troponin T (cTnT) is considered as a highly sensitive and specific marker of myocardial damage.[5] Cardiac troponins are regulatory proteins of the thin actin filaments of the cardiac muscle. Troponin T and troponin I are highly sensitive and specific markers of myocardial injury. Cardiac troponins are detected in serum or heparin plasma using monoclonal antibodies against several different epitopes of the troponin T or I molecule. These antibodies have negligible cross-reactivity to skeletal muscle.[6]

Acute valvular disease with heart failure or chronic valvular disease account for most of the morbidity and mortality associated with rheumatic fever.[7] Revised guidelines for the diagnosis of rheumatic fever indicate that when rheumatic fever affects the heart, it usually involves the endocardium, myocardium, and pericardium to varying degrees. These guidelines also indicate that rheumatic myocarditis, although “uncommon” in the absence of severe valvular damage, “may contribute” to the genesis of heart failure during rheumatic fever.[8] However, the existence of a specific primary myocardial involvement contributing to the occurrence of heart failure during rheumatic fever is controversial and still needs further studies.

The aim of this study was to investigate the role of myocardial involvement in the hemodynamic changes in patients with acute rheumatic fever using echocardiography and cTnT as a specific marker of myocardial damage.

## PATIENTS AND METHODS

The study was a prospective cohort study conducted on the Suez Canal University Hospital.

### Inclusion criteria

Children admitted with the diagnosis of acute rheumatic fever according to the modified Jones criteria.[9]

### Exclusion criteria

It includes the presence of associated congenital heart disease and recurrent or chronic rheumatic valvular lesions. For each patient, the following was done: complete history and physical examination to detect inclusion and exclusion criteria. A blood sample was collected from each patient to measure ESR, C-reactive proteins, ASOT, and cTnT. Electrocardiography and echocardiography were performed for each child. The patients received the regular treatment as per the hospital protocol for the cases of rheumatic fever. The patients were divided into two groups according to the presence or absence of carditis. Carditis was defined clinically as the prolongation of PR interval in the ECG, sleeping tachycardia out of proportion of fever, the presence of a new murmur of aortic or mitral regurgitation or the presence of heart failure. This was confirmed by echocardiography at the time of the diagnosis.[10]

### Cardiac troponin T assessment

A sample of blood was taken from each child. The blood was centrifuged, separated, and frozen at –20 °C until batch analysis was performed. Biochemical analysis was performed with an Elecsys Troponin T STAT Immunoassay (Roche Diagnostics GmbH, Mannheim, Germany). This electrochemiluminescent sandwich enzyme-linked immunosorbent assay has a lower limit of detection of 0.01 ng/mL, and the normal range for cTnT is 0.01–0.1 ng/mL.

### Echocardiographic assessment

It was performed using the Hewlett-Packard sonos 1800 series equipped with 3.5–5 MHz transducer. All studies included parasternal long and short axis views, apical four, five chamber views, subxiphoid four chamber, and suprasternal view. Ejection fraction and fractional shortening were determined, and the severity of valvular regurgitation was graded qualitatively from 0 to 4 (0: absent, 1: trivial to mild, 2: moderate, 3: moderate to severe, and 4: severe)[10]

### Statistical analysis

The data were analyzed using the statistical package SPSS. Descriptive statistics were used to present the characteristics of the study population. Continuous data were compared using Student’s t-test. Categorical data were compared with a two-tailed c2 test. Statistical significance was accepted as P < 0.05.

### Ethical consideration

A written consent was given from participants’ family with approval of ethics committee.

## RESULTS

The study group comprised 34 children with ARF, 19 (56%) were females and 15 (44%) were males. The mean age of the study group was 11.3 ± 2.65 years.

Out of the study population, 20 children (59%) showed evidence of carditis, and 14 children (41%) had ARF without evidence of carditis.

Table 1 shows the characteristics of the study population. From this table, we can see that there is no significant difference between the two groups of children (with and without carditis) regarding the age, sex, CRP, and ESR. The PR interval on ECG was prolonged in the group of patients with carditis (0.17 ± 0.03 s) compared to the group without carditis (0.10 ± 0.05 s). The difference was statistically significant. In addition, the resting pulse rate was significantly higher in the group of patients with carditis (109.3 ± 15.7 beats/min), compared to those without carditis (89.6 ± 19.4 beats/min).

**Table 1:** Clinical and biological characteristics of the study groups

	ARF with carditis, n = 20 (59%)	ARF without carditis, n = 14 (41%)	*P*
Age (years)	11.5 ± 4.6	10.9 ± 5.3	0.374
Sex			
Female	12	8	0.083
Male	8	6	
CRP (mg/dL)	6.5 ± 5.2	7.1 ± 4.9	0.153
ESR (mm/h)	69 ± 21.3	58 ± 26.8	0.215
PR (s)	0.17 ± 0.03	0.10 ± 0.05	0.021*
Heart rate (b/min)	109.3 ± 15.7	89.6 ± 19.4	0.013*

* Significant difference.

The distribution of valvular lesions in children with carditis is shown in Table 2. Fifteen children had isolated mitral regurgitation (four children grade 1, seven children grade 2, and four children grade 3). Another three patients had both mitral and aortic regurgitation (grade 2). Isolated aortic regurgitation was present in two children (one child grade 1, and one child grade 2). Among the 20 children with carditis, heart failure was present in five children with mitral regurgitation.

**Table 2:** Distribution of valvular lesions among children with carditis

	Grade 1 (trivial to mild)	Grade 2 (moderate)	Grade 3 (moderate to severe)	Grade 4 (severe)
MR (n)	4	7	4	0
AR (n)	1	1	0	0
MR + AR (n)	0	3	0	0

MR: Mitral regurgitation; AR: Aortic regurgitation; N: Number of children.

Table 3 shows the cTnT level in both groups of children with ARF. The mean level of cTnT in patients with carditis is 0.051 ± 0.01 ng/mL, while the mean level of cTnT in patients without carditis is 0.039 ± 0.02 ng/mL. The difference is not significant (*P* > 0.05).

**Table 3:** Cardiac troponin T level in the study groups

	ARF with carditis, *n* = 20 (59%)	ARF without carditis, *n* = 14 (41%)	*P*
Troponin T (ng/dL)	0.051 ± 0.01	0.039 ± 0.02	0.089

Table 4 shows the echocardiographic measurements in the two groups of children with ARF. We can see from this table that the left ventricular ejection fraction is 59.2 ± 7.3% in patients with carditis and 65.6 ± 6.4% in patients without carditis. The difference is not significant (*P* > 0.05). In addition, there was no significant difference between the two groups regarding the fractional shortening which was 31.8 ± 6.2% in the group of children with carditis and 37.5 ± 6.4% in the children without carditis (*P* > 0.05).

**Table 4:** Functional echocardiography data

	ARF with carditis, *n* = 20 (59%)	ARF without carditis, *n* = 14 (41%)	*P*
Ejection fraction (%)	59.2 ± 7.3	59.2 ± 7.3	0.087
Fractional shortening (%)	31.8 ± 6.2	37.5 ± 6.4	0.216

## DISCUSSION

Despite pathologic evidence of myocardial inflammation, the significance of myocarditis in children with acute rheumatic carditis remains controversial.[11] The guidelines for the diagnosis of rheumatic fever indicated that rheumatic myocarditis, although uncommon, in the absence of severe valvular damage may have contributed to the genesis of heart failure during rheumatic fever.[8]

In this study, we could not find any biochemical alteration or echocardiographic measurements change suggestive of significant hemodynamic effects due to myocardial involvement. No statistically significant difference was found in the level of cTnT between patients with and without rheumatic carditis. Furthermore, all patients showed cTnT level within the normal range even in those with heart failure. Cardiac troponin T is considered as one of the new “gold markers” of ischemic myocardial injury and has been detected as a sensitive and specific marker of even subclinical myocardial injury. It is also used in the diagnosis and monitoring of nonischemic myocardial injury, like myocarditis.[12]

In addition, the echocardiographic study failed to show any significant difference in the left ventricular ejection and shortening fractions between children with and without rheumatic carditis whether or not they had congestive heart failure. Our results are in agreement with Tavli *et al*.,[13] who showed that the level of cTnI is not elevated in acute rheumatic fever in children with and without carditis. In addition, they failed to show any echocardiographic signs of myocardial involvement in their study on rheumatic fever in children. Alehan *et al*.[14] also demonstrated that serum cTnT concentration does not increase above normal limits in rheumatic carditis with or without heart failure, also they could not find any significant change in function due to myocarditis using echocardiography. Kamblock *et al*.[15] examined a large series of children with ARF (95 children), they neither detected cTnI elevations nor echocardiographic abnormalities suggesting significant hemodynamic changes due to myocardial involvement during rheumatic fever, in patients with carditis even if they had congestive heart failure. Congestive heart failure was always related to severe valve regurgitation not to myocarditis. The same results were shown by Williams *et al*.,[4] who demonstrated that serum cTnI was not elevated in patients with acute rheumatic fever. They stated that the fact that levels of cardiac troponin I are not elevated in the serum of children with acute rheumatic carditis suggesting that there is minimal myocyte necrosis in this setting.

The low cTnT values, especially in the presence of active carditis, dispute significant ischemic myocyte injury.[16] Myocardial necrosis is not prominent despite intensive inflammation in ARF.[17] ARF is mainly a disease of connective tissue, and endocarditis is the prominent factor.[18] This may explain the normal concentrations of cTnT found in patients with ARF carditis. Aschoff nodules are the histological hallmark of rheumatic fever, and have been identified in left atrial appendages and ventricular myocardium of patients with rheumatic fever and rheumatic heart disease.[19] However, the presence of Aschoff nodules in the ventricular myocardium does not signify that they play a role in the genesis of congestive heart failure during the acute stage of the disease.[8]

We can conclude that this study did not demonstrate an elevated cTnT level suggesting clinically relevant myocardial involvement during acute rheumatic fever even in patients with severe carditis and congestive heart failure. In addition, we failed to show significant difference in echocardiographic measurements between patients with and without carditis. These findings substantiate the view that myocardial involvement during acute rheumatic fever has no significant hemodynamic effects and does not play a role in the development of heart failure in patients with rheumatic carditis. Heart failure is related mainly to the extent of valvular lesions rather than the myocardial involvement.

However, among study limitations are rather small number of studied population. Further studies are needed to confirm that myocardial involvement during acute rheumatic fever is not relevant in the development of heart failure in patients with rheumatic fever.
